# “I can’t remember the last time I was comfortable about being home”: lived experience perspectives on thriving following homelessness

**DOI:** 10.1080/17482631.2023.2176979

**Published:** 2023-02-20

**Authors:** Carrie Anne Marshall, Brooke Phillips, Julia Holmes, Eric Todd, River Hill, George Panter, Corinna Easton, Terry Landry, Sarah Collins, Tom Greening, Ashley O’Brien, Marlo Jastak, Rebecca Ridge, Rebecca Goldszmidt, Chelsea Shanoff, Debbie Laliberte Rudman, Alexandra Carlsson, Suliman Aryobi, Jessica Szlapinski, Rozelen Carrillo-Beck, Nicole Pacheco, Shauna Perez, Abe Oudshoorn

**Affiliations:** a Social Justice in Mental Health Research Lab, School of Occupational Therapy, Western University, London, Canada; b Providence Care Hospital, Kingston, Canada; c Salvation Army London Centre of Hope, London, Canada; d Home Base Housing, Kingston, Canada; e Trellis HIV and Community Care, Kingston, Canada; f Addiction and Mental Health Services Kingston, Frontenac, Lennox & Addington (KFLA), Kingston, Canada; g School of Occupational Therapy, Western University, London, Canada; h School of Nursing, Western University, London, Canada

**Keywords:** Social justice, health equity, housing, health systems, poverty, community integration, meaningful activity, mental health, substance use

## Abstract

**Purpose:**

Strategies for preventing and ending homelessness are frequently measured by their effectiveness on indices of tenancy sustainment. To shift this narrative, we conducted research to identify what is needed to “thrive” following homelessness from the perspectives of persons with lived experience in Ontario, Canada.

**Methods:**

Conducted in the context of a community-based participatory research study aimed at informing the development of intervention strategies, we interviewed 46 persons living with mental illness and/or substance use disorder [*n* = 25 (54.3%) unhoused; *n* = 21 (45.7%) housed following homelessness] using qualitative interviews. A subsample of 14 participants agreed to engage in photovoice interviews. We analysed these data abductively using thematic analysis informed by health equity and social justice.

**Results:**

Participants described experiences of “living in a state of lack” following homelessness. This essence was expressed through four themes: 1) housing as part one of the journey to home; 2) finding and keeping “my people”; 3) meaningful activity as critical for thriving following homelessness; and 4) struggling to access mental health supports in the context of challenging circumstances.

**Conclusions:**

Individuals struggle to thrive following homelessness in the context of insufficient resources. There is a need to build on existing interventions to address outcomes beyond tenancy sustainment.

## Introduction

Widening income inequality, a lack of investment in public housing, and a housing market that has become rapidly unaffordable in recent decades have converged to result in a growing homelessness crisis in most high-income countries internationally leaving millions to experience homelessness every year [Australian Institute of Health and Welfare (AIHW), 2019; Baptista & Marlier, 2019; National Alliance on Ending Homelessness (NAEH), 2020]. It is widely acknowledged that persons living with mental illness and substance use disorders are disproportionately and systematically affected by homelessness (Fazel et al., 2014; Hossain et al., 2020). In one systematic review, the overall prevalence of mental illness amongst persons experiencing homelessness was as high as 77.5%, and up to 60.9% for substance use disorder (Hossain et al., 2020). Further, persons living with mental illness and/or substance use disorder are known to experience high rates of poverty (Forchuk & Csiernik, 2021), thereby amplifying the risk of homelessness. These high prevalence rates, combined with the many other comorbid health conditions experienced by this population (Fazel et al., 2014) has led some scholars to declare homelessness as a disability-rights violation (Farha, 2015). The undignified conditions in which individuals live during homelessness only deepens the disability and health inequities they experience (Gaetz et al., 2016; NAEH, 2021; Pleace et al., 2013) and a growing body of literature suggests that some of these challenges continue following homelessness, thereby interfering with tenancy sustainment and well-being (Boland et al., 2021; Kerman & Sylvestre, 2020). There is a need to invest in solutions that not only immediately end homelessness, but also prevent ongoing homelessness by supporting individuals to attain the conditions that support optimal well-being after securing a tenancy.

### Living conditions during and following homelessness

During homelessness, persons living with mental illness and substance use disorders are exposed to inadequate living conditions, trauma, and deep degrees of discrimination and stigma (Carrillo Beck et al., 2022; Jensen, 2017). These social conditions worsen the experience and severity of mental illness and substance use disorder and prolong homelessness (AIHW, 2019; Baptista & Marlier, 2019; NAEH, 2020). Combined with a dire lack of deeply affordable housing, this creates a situation in which many individuals who live with mental illness and in low income are unhoused for many years of their lives (Piat et al., 2014). Providing immediate and permanent housing has the potential to limit exposure to many of the conditions that contribute to poorer health in this population; however, recent research indicates that while housing is absolutely necessary, many individuals continue to experience ongoing challenges with attaining the conditions that enable a basic degree of well-being following homelessness (C.A. Marshall et al., 2022; 2020; 2022). Such challenges include ongoing poverty, food insecurity (Easton et al., 2022), poor community integration and loneliness (Dej, 2020; Marshall, et al., 2020; Quilgars & Pleace, 2016). In one systematic review that included 57 studies conducted in high-income countries, the conditions in which individuals were situated following homelessness often led to participants worrying about their ability to sustain their tenancies (C. A.Marshall et al., 2022). While solutions for ending homelessness are known, and have demonstrated effectiveness, there is a need to build on these solutions to prevent ongoing homelessness and support well-being after homelessness ends.

### Evidence-based interventions for addressing homelessness

Strategies developed to support persons who experience homelessness have focused primarily on the security and maintenance of a tenancy. Historically, such services were informed by the belief that individuals leaving homelessness must have their mental health, substance use, and other challenges addressed before they could be successful in sustaining permanent housing (O’Shaughnessy & Greenwood, 2021). Such services have come to be known as “staircase supports” (SS) or “treatment first” approaches, and emphasize the use of shelters, transitional housing, and abstinence-based services designed to encourage “housing readiness”. SS have long been criticized for creating undignified circumstances that neglect the needs of individuals by denying the right to permanent housing, and have been deemed to be largely ineffective for improving housing and well-being outcomes (Denvall et al., 2022). These criticisms have led to widespread abandonment of SS in recent years (Denvall et al., 2022). Instead, other approaches including “Critical Time Intervention” (CTI) and “Housing First” (HF) have since been adopted in place of SS (Ponka et al., 2020; Tsemberis, 2010).

Both CTI and HF have consistently demonstrated greater effectiveness over SS for supporting individuals to secure a tenancy and stay housed for longer (Ponka et al., 2020; Stergiopoulos et al., 2014; Tsemberis, 2010). HF, however, has been the subject of a far greater number of effectiveness studies, and its consistent performance in improving tenancy sustainment is the reason for its wide adoption across a large majority of communities in North America and Europe (Gaetz et al., 2016; Pleace, 2016). When delivered as designed, HF represents a dignified approach to supporting individuals who live in housing precarity because it emphasizes the right to housing without any preconditions and is fundamentally person driven. HF is a critical, evidence-based intervention that needs to continue to be implemented; however, it should not be regarded as a panacea (C. A. Marshall et al., 2020; Quilgars & Pleace, 2016). Recent research suggests that many individuals living with mental illness and/or substance use difficulties have a variety of ongoing unmet psychosocial needs after leaving homelessness, even when they receive HF as an intervention. These include ongoing poverty (Gaetz et al., 2016), low levels of community integration (Raphael-Greenfield & Gutman, 2015), high levels of substance use (Somers et al., 2015), ongoing symptoms of mental illness (Gaetz et al., 2016), low levels of engagement in meaningful activity (C. A. Marshall et al., 2018, 2020), and food insecurity (Parpouchi et al., 2016). Systems that allow for poor adherence to the HF model, that are poorly integrated, that do not adequately account for consumer choice (Oudshoorn, Smith-Carrier, et al., 2021), or that target the security and maintenance of a tenancy as a primary indicator of program effectiveness, may contribute to these outcomes. Still, even when HF is implemented with a high degree of fidelity, research indicates that while housing outcomes are well addressed, effectiveness on psychosocial outcomes continues to be mixed or poor (Goering et al., 2014; Woodhall-Melnik & Dunn, 2015). New approaches that build on HF and other existing supports are needed to enable individuals to not only sustain their tenancies after leaving homelessness, but to thrive in their communities after securing a tenancy.

### The current study and associated theoretical framework

For good reason, much of the literature on homelessness is focused on experiences of homelessness and identifying strategies for supporting individuals to secure and sustain a tenancy. Fewer studies have identified the conditions needed to “thrive” following homelessness from the perspectives of persons with lived experience; namely, conditions for both sustaining one’s tenancy, and attaining necessary conditions for optimal well-being (Boland et al., 2018; Oudshoorn, Van Berkum, et al., 2021). Existing interventions which are known to be effective for tenancy sustainment such as CTI and HF, have failed to demonstrate effectiveness for improving indices of psychosocial well-being following homelessness (Goering et al., 2014; Quilgars & Pleace, 2016). Attending to more than tenancy sustainment alone is necessary for improving psychosocial outcomes following homelessness and may be a key homelessness prevention strategy.

Guided by the lenses of social justice (Jost & Kay, 2010) and health equity (Sen et al., 2004), we conducted a qualitative study aimed at identifying what is needed to enable thriving following homelessness within the current system of supports in two cities in Ontario, Canada (Kingston & London). This research was designed to generate data that could be used as a foundation to co-design solutions for promoting thriving following homelessness. Social justice and health equity theories were recognized as useful lenses through which to conduct this research given that our primary concerns were to identify social injustices experienced by individuals after leaving homelessness and advocate for improvements in current supports to enable thriving rather than survival alone. As such, this research was guided by the question: What are the strengths and challenges of existing supports to enable thriving following homelessness from the perspectives of persons with lived experience in two communities in Ontario, Canada?

## Methodology

This paper presents qualitative findings from a community-based participatory research (CBPR) (Hacker, 2013) study aimed at identifying what is needed to build on existing services to better enable thriving following homelessness. This was followed by using the information gathered to co-design a novel intervention to compliment existing services. The parent study, called the “Transition from Homelessness Study,” involved qualitative interviews with: 1) persons living with mental illness and/or substance use disorder with experiences of homelessness; and 2) service providers and organizational leaders working in housing and homelessness services. Findings detailing the perspectives of service providers and organizational leaders in this research and our co-design process can be found in two separate papers (C. A. Marshall et al., 2022; Marshall, Oudshoorn, et al., 2022). Methods and findings associated with lived experience interviews are detailed in this paper.

### Recruitment

After receiving ethics approval from both Western and Queen’s Universities, we purposively recruited persons with experiences of homelessness from community organizations serving persons who are unhoused or housed following homelessness in Kingston and London, ON, Canada. Participants were recruited in each recruitment city from shelters, permanent supportive and transitional housing programs, and urban encampments. In order to recruit participants from these settings, we: 1) sent emails directly to leaders of health and social care organizations detailing information about our study and requested their support with recruitment; 2) presented to shelter and case management staff within relevant organizations and encouraged service providers to provide contact information for the research team directly to potential participants; 3) attended local organizations for drop-in times during which interested individuals could participate and ask questions about our study; and 4) accompanied outreach staff in urban encampments, where we recruited participants directly. In our recruitment efforts, we attempted to include a diverse group of participants based on housing status, age and gender.

#### Inclusion and exclusion criteria

We included participants who: 1) were over the age of 16; 2) were either unhoused or housed following homelessness; and 3) acknowledged living with a mental illness and/or substance use disorder. Unhoused participants were included if they had been unhoused for at least one month in their current episode of homelessness. Housed participants were included if they had experienced at least one month of homelessness within the past three years.

### Procedure

#### Semi-structured qualitative interviews

We arranged suitable times and dates with individuals meeting inclusion criteria to facilitate the conduct of interviews. Once an interview was arranged, a member of our research team (CM, BP, ET, GP, RG, CS, AO) met with participants in a private interview space within the buildings of collaborating organizations, or a private area situated within an urban encampment. Interviewers began by reading aloud a letter of information requesting consent to participate in interviews. Once participants consented, we posed demographic questions, which were recorded by a member of our research team on a tablet computer through a survey developed in Qualtrics (2018). All interview questions, letter of information and consent were read aloud to participants to overcome threats to trustworthiness associated with the potential for low literacy. Demographic questions in the survey included: age; gender; sexual orientation; race and ethnicity; income; self-reported mental health conditions; substance use measured by the Alcohol Use Disorders Identification Test (AUDIT-10) (Babor et al., 2001) and the Drug Abuse Screening Test (DAST) (Skinner, 1982); and housing status. At this time, participants were asked to identify a pseudonym to protect their confidentiality, which was used to refer to quotes in the findings section of this paper.

**Figure 1.**
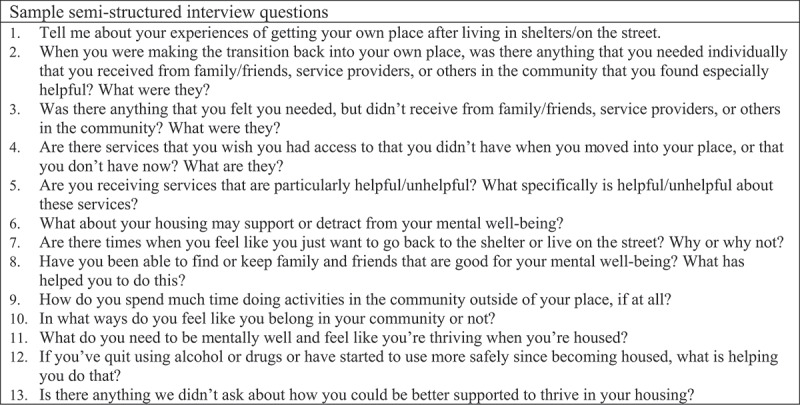
Sample semi-structured interview questions.

Once participants had provided demographic information, they were engaged in semi-structured interviews using a qualitative interview guide. Questions posed to participants focused on identifying the strengths and challenges associated with existing supports in their respective community (Kingston or London, ON), and their perspectives on what is needed to thrive following homelessness. Participants were provided $40 for participating in these interviews. Interviews were recorded on a digital recording device and transcribed verbatim. Sample interview questions posed to participants are provided in Figure 1.

#### Photovoice interviews

Following initial interviews, a sub-sample was approached to engage in photovoice interviews aimed at generating a visual representation of what is needed for thriving following homelessness within the current system of available services. We purposively approached participants representing diversity in housing status, age and gender to participate in these interviews. Photovoice is a form of auto photography in which participants interviewed in the context of participatory research are asked to provide photographs depicting changes needed in their community to improve their lives (Glaw et al., 2017; Wang & Burris, 1997). These photographs, and the descriptions provided by participants, are then used to advocate for changes to community services and current policies (Glaw et al., 2017; Wang & Burris, 1997).

Participants who consented were individually provided with training on the purpose of taking photographs, and ethical procedures for photographing others and in public spaces. To protect the safety and confidentiality of both participants and community members, each participant was provided with a photo release form for community members to sign if any identifying information was photographed pertaining to them. Participants were each provided with a digital camera or opted to use their personal cell phone. Appointments were made with participants to meet one week following initial interviews to share their photographs with a member of our research team. During this second meeting, participants were asked to describe each photograph in relation to the research question. These meetings were video recorded to enable our team to understand the meaning of each photograph to participants, resulting in an audio-visual interview. Because we did not want the photographs to be separated from participant narratives, we did not transcribe these interviews in full. We did, however, transcribe selected participant descriptions of photographs to ensure that our interpretation of each was derived directly from the narratives of participants. Participants were provided with $200 as compensation for taking photographs and for meeting in this second interview.

### Analysis

We analysed interview transcripts abductively informed by theories of social justice (Jost & Kay, 2010) and health equity (Sen et al., 2004) using thematic analysis (Braun & Clarke, 2014). Using Dedoose, a cloud-based qualitative data management program that facilitated the organization of our data (SocioCultural Research Consultants, 2015), several members of our team (CM, BP, JH, CE, RG) coded statements pertaining to the research question, followed by grouping these codes into like categories. These categories were then arranged into themes. These themes were refined through several discussions among the coders. Consistent with the method proposed by Braun and Clarke, a central essence that characterized participant interviews was identified among the research team (Braun & Clarke, 2014). Photovoice interviews were used to provide additional visual context to the findings of our semi-structured interviews with the full participant sample, and as such, were discussed throughout our analysis. As we analysed interviews from the full sample, we regularly referred to these photographs, and discussed participant interpretations of each. As our analysis progressed, photographs that visualized our themes were chosen, and participant narratives describing each photograph were transcribed and paired. Once our findings were analysed and written, final feedback was provided by all study authors on the analysis presented which included both data from semi-structured and photovoice interviews. This feedback was then used to refine our analysis further.

To evaluate the rigour of our methodology, we compared our approach against the Standards for Reporting Qualitative Research (SRQR) (O’Brien et al., 2014) and the Consolidating Criteria for Reporting Qualitative (COREQ) research (Booth et al., 2007) checklists. All criteria on the SRQR checklist were met, however, criteria on the COREQ checklist were met with the exception of: 1) taking field notes due to the fact that our team was large and field notes may have varied substantially across interviewers; 2) returning transcripts to participants for comment or correction; and 3) participant/member checking. The latter two criteria were not met as we did not feel confident that given that nature of the sample, that we would have been able to locate participants following interviews.

#### Trustworthiness

Strategies for establishing trustworthiness identified by Lincoln and Guba (1985) were used throughout the process of this study, and included: (a) prolonged engagement with the population of interest, which was achieved through the research team’s extensive lived experience, involvement in research and practice related to homelessness, and pre-existing relationships with the recruitment organizations; (b) peer debriefing, which involved continuous debriefing among several members of the research team involved in data collection and analysis; (c) recording interviews; (d) accurate transcription; (e) triangulation, which was achieved through incorporating data from multiple sources (semi-structured and photovoice interviews); (f) intercoder consensus; and (g) use of a computer program to organize data (Dedoose), which contributed to the dependability of our analysis.

#### Reflexivity

In conducting our analysis, we have assumed a constructivist epistemology recognizing that participants and researchers construct their own views through the lenses of their prior experiences and through interactions with each other (Charmaz, 2014; Denzin & Lincoln, 2011). Together, the principal investigator and several members of our research team have decades of combined lived experiences of homelessness, conducting research related to homelessness, and practice in a range of health and social care professions with individuals during and following homelessness. We recognize the impossibility of setting aside any preconditions to analyse our data. Instead, we have embraced this knowledge as a strength in informing our analysis and have been explicit regarding our theoretical influences.

## Findings

### Sample characteristics

We interviewed 46 participants composed of *n* = 26 (56.5%) men, *n* = 19 (41.3%) women, and *n* = 1 (2.2%) participant identifying as non-binary. Participants resided in Kingston, Ontario, Canada (*n* = 19; 41.3%) and London, Ontario, Canada (*n* = 27; 58.7%) and ranged in age from 17–68 (Mdn = 41; IQR = 21) years old. Of the full sample, *n* = 25 (54.3%) participants were unhoused, and *n* = 21 (45.7%) were housed following homelessness. The majority of the sample was White (*n* = 35; 76.1%), and heterosexual (*n* = 37; 80.4%). Participants were largely unemployed and supported primarily by income support programs including disability-related social assistance [Ontario Disability Support Program (ODSP)] (*n* = 27; 58.7%) and general social assistance [Ontario Works (OW)] (*n* = 13; 28.3%). The most common forms of mental illness endorsed by participants were mood disorder (*n* = 40; 87%), anxiety disorder (*n* = 38; 82.6%) and stress and trauma-related disorders (*n* = 36; 78.2%). The majority of participants reported non-hazardous alcohol use on the AUDIT-10 (*n* = 35; 76.1%), and over half were engaged in substance use that was moderate or less (*n* = 28; 60.8%). Of this sample, 14 participants engaged in photovoice interviews. Participants in this sub-sample consisted of *n* = 9 (64.3%) men and *n* = 5 (35.7%) women, of whom *n* = 7 (50%) were unhoused, and *n* = 7 (50%) were housed. A complete summary of the demographic characteristics of participants is provided in Table 1. Housing and health characteristics of participants are summarized in Table 2.

**Table I.** Lived experience participant characteristics (*n* = 46).

Demographic Characteristics			
	Kingston (n = 19) n (%)	London (n = 27) n (%)	Full Sample (n = 46) n (%)
Gender			
Men	12 (63.2)	14 (51.9)	26 (56.5)
Women	6 (31.6)	13 (48.1)	19 (41.3)
Non-binary	1 (5.3)	-	1 (2.2)
Age	(23–68; Mdn = 42; IQR = 30)	(17–68; Mdn = 40; IQR = 16)	(17–68; Mdn = 41; IQR = 21)
Sexual orientation			
Heterosexual	15 (78.9)	22 (81.5)	37 (80.4)
Lesbian	-	-	-
Gay	-	-	-
Bi-sexual	2 (10.5)	4 (14.8)	6 (13.0)
Transsexual	1 (5.3)	1 (3.7)	2 (4.3)
Queer	1 (5.3)	-	1 (2.2)
Two-spirit	-	-	-
Race/Ethnicity			
White	15 (78.9)	20 (74.1)	35 (76.1)
Black	-	2 (7.4)	2 (4.3)
First Nations	3 (15.8)^1^	-	3 (6.5)
Metis	1 (5.3)	-	1 (2.2)
Hispanic	-	1 (3.7)	1 (2.2)
Mixed Race	-	3 (11.1)	3 (6.5)
Prefer not to answer	-	1 (3.7)	1 (2.2)
Income source			
ODSP	13 (68.4)	14 (51.9)	27 (58.7)
OW	4 (21.1)	9 (33.3)	13 (28.3)
CPP	2 (10.5)	1 (3.7)	3 (6.5)
OAS	1 (5.3)	1 (3.7)	2 (4.3)
Student loan	2 (10.5)	-	
Long term disability through former employer	-	1 (3.7)	1 (2.2)
Employment	1 (5.3)	4 (14.8)	5 (10.9)
Self-employment	2 (10.5)	1 (3.7)	3 (6.5)
Panhandling	7 (36.8)	-	7 (15.2)
Bottle collecting	4 (21.1)	-	4 (8.7)
Scrap metal collection	2 (10.5)	-	2 (4.3)
Shovelling snow	1 (5.3)	-	1 (2.2)
Sex work	1 (5.3)	-	1 (2.2)
Selling substances	3 (15.8)	-	3 (6.5)

ODSP=Ontario Disability Support Program; OW=Ontario Works; CPP=Canada Pension Plan; OAS=Old Age Supplement.

^a^(Cree = 1; Mohawk = 1; Ojibwa = 1).

**Table II.** Participant housing history and health status (*n* = 46).

Characteristic			
	Kingston (n = 19) n (%)	London (n = 27) n (%)	Full Sample (n = 46) n (%)
Housing situation at the time of the interview			
Unhoused	10 (52.6)	15 (55.6)	25 (54.3)
Have you been unhoused for one year continuously in the past three years?			
Yes	4 (21.1)	9 (33.3)	13 (28.3)
No	6 (31.6)	6 (22.2)	15 (32.6)
Unknown/cannot recall	9 (47.4)	12 (44.4)	21 (45.7)
Sleep location			
Shelters	8 (42.1)	14 (51.9)	22 (47.8)
Outdoors	8 (42.1)	1 (3.7)	9 (19.6)
Couch-surfing	4 (21.1)	2 (7.4)	6 (13.0)
Motels	1 (5.3)	-	1 (2.2)
Hospital waiting room	1 (5.3)	-	1 (2.2)
Warming centre	1 (5.3)	-	1 (2.2)
Housed following homelessness	9 (47.4)	12 (44.4)	21 (45.7)
How many months have you been housed?	(1–35; Mdn = 6.5; IQR = 27)	(6–36; Mdn = 13; IQR = 9)	(1–36; Mdn = 12; IQR = 16)
Months unhoused before housing	(1–48; Mdn = 12.5; IQR = 19)	(3–54; Mdn = 12; IQR = 34)	(1–54; Mdn = 12; IQR = 22)
Housing type			
PSH (Cluster site)	3 (15.8)	1 (3.7)	4 (8.7)
PSH (Scatter site)	-	1 (3.7)	1 (2.2)
Social housing	-	6 (22.2)	6 (13.0)
Market rent unit	6 (31.6)	4 (14.8)	10 (21.7)
Missing	10 (52.6)	15 (55.6)	25 (54.3)
At what age did you first experience homelessness?	(14–57; Mdn = 15; QR = 24)	(13–68; Mdn = 25; IQR = 20)	(13–68; Mdn = 25; IQR = 21)
Frequency of tenancy loss in the past three years			
Unhoused for the past three years	-	4 (14.8)	4 (8.7)
Never	4 (21.1)	-	4 (8.7)
One time	1 (5.3)	8 (29.6)	9 (19.6)
Two times	3 (15.8)	2 (7.4)	5 (10.9)
Three times	1 (5.3)	-	1 (2.2)
More than three times	1 (5.3)	1 (3.7)	2 (4.3)
Unknown/cannot recall	9 (47.4)	12 (44.4)	21 (45.7)
Mental health conditions			
Mood disorder	17 (37.0)	23 (50)	40 (87.0)
Anxiety disorder	17 (37.0)	21 (45.7)	38 (82.6)
Stress and trauma disorder	17 (37.0)	19 (41.3)	36 (78.2)
Obsessive-compulsive disorder	9 (19.6)	7 (15.2)	16 (34.8)
Psychotic disorders	7 (15.2)	7 (15.2)	14 (30.4)
Personality disorder	7 (15.2)	5 (10.9)	13 (28.3)
Eating disorder	1 (2.2)	-	1 (2.2)
Substance Use			
Alcohol use (AUDIT)	0–40 (Mdn = 2; IQR = 2)	0–31 (Mdn = 1; IQR = 7)	0–40 (Mdn = 1; IQR = 7)
Non-hazardous use (˂8)	14 (30.4)	21 (45.7)	35 (76.1)
Hazardous use (≥8)	5 (10.9)	6 (13.0)	11 (23.9)
Drug use (DAST)	0–9 (Mdn = 4; IQR = 7)	0–10 (Mdn = 4; IQR = 8)	0–10 (Mdn = 4; IQR = 7)
No problem (0)	6 (13.0)	12 (26.1)	18 (39.1)
Low level (1–2)	1 (2.2)	1 (2.2)	2 (4.4)
Moderate level (3–5)	5 (10.9)	3 (6.5)	8 (17.4)
Substantial level (6–8)	6 (13.0)	7 (15.2)	13 (28.3)
Severe level (9–10)	1 (2.2)	4 (8.7)	5 (10.9)

PSH = Permanent supportive housing; AUDIT = Alcohol Use Disorders Identification Test; DAST = Drug Abuse Screening Test; Mdn = Median; IQR = Interquartile range.

### Qualitative findings

**Figure 2.**
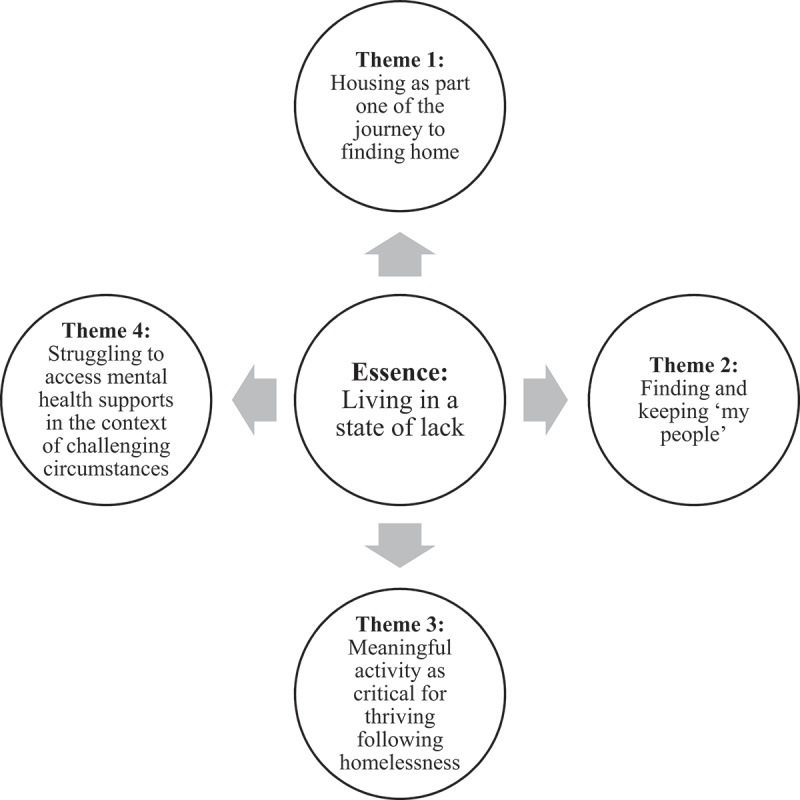
Theme structure.

Semi-structured interviews ranged from 9–85 minutes (Mdn = 35; IQR = 19.3). The overarching essence of interviews was “living in a state of lack”. This essence was informed by four themes generated in our analysis: 1) housing is part one of the journey to finding home; 2) finding and keeping “my people”; 3) meaningful activity as critical for thriving following homelessness; and 4) struggling to access mental health supports in the context of challenging circumstances. A visual depiction of this theme structure is provided in Figure 2, and the essence and associated themes are described below.

#### Essence: living in a state of lack

The overarching essence expressed through the themes generated in our analysis was that participants found it difficult or impossible to secure the conditions necessary to move beyond homelessness and thrive in their communities after. Participants discussed living constantly without basic resources for survival including adequate housing, income, and access to nutritious food following homelessness. They described being in a constant struggle to survive and identified that this experience negatively impacted their mental health. This situation was complicated by a lack of good quality, affordable housing in the cities in which they resided, causing them to choose the first housing option available to them, whether it met their needs or not:

Believe me. People are so dire. We’re right on the onset of winter here. People are so desperate to get off the street and get away from the cold that they’ll dive at anything. Even the kind of situation that I am in. I’m almost forced into situations like this … because of low income and unavailable housing. We’re forced to live in rooms with other people. We’re forced to do that. It’s not a nice word to use, but if you think about it all, it leaves us absolutely no choice. And when you’re already feeling as helpless and homeless … then having that happen makes it all much worse. [Pekoe, Housed]

Participants indicated that the state of this system prevented them from sustaining their housing and thriving after leaving homelessness. They recognized that these issues transcended local services and needed to be addressed at the macro (structural) level. This situation led to feelings of frustration and abandonment, as they knew exactly what was needed to prevent ongoing homelessness and to thrive in their communities after:

We strive to find shelter. We strive to find food. And we just want a place to live and more money from the government. That’s the end. [Donny, Unhoused].

#### Theme 1: housing as part one of the journey to finding home

Participants described the importance of housing for their well-being; however, they also recognized that housing alone did not create a sense of “home”. They emphasized that living in a state of survival on an ongoing basis prevented them from attaining thriving. Housing that was available was described as unsafe and of low quality in that it was poorly maintained, and at times, infested with vermin, bedbugs and cockroaches. This created a situation in which the housing that was available was far less comfortable than living in shelter spaces or on the street. When offered such an apartment to rent during homelessness, Casey recalled: “*I chose homelessness over living there*” [Casey, Housed]. Participants who were housed indicated that they had endured housing that was of such poor quality that: “*I can’t remember the last time I was comfortable about being home*” [Matt, Unhoused].

**Figure 3.**
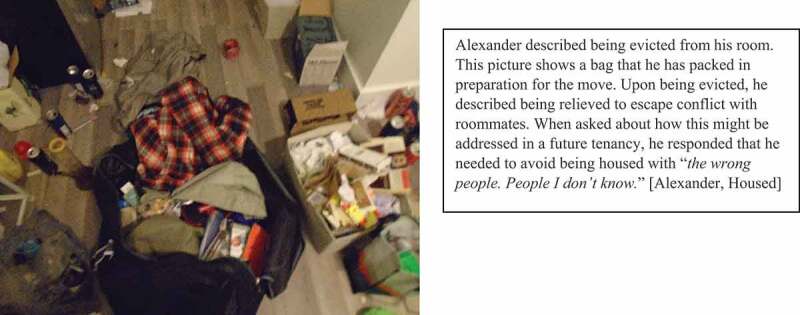
Evicted.

To afford housing, many participants chose shared accommodation as private units were often unaffordable. Living in close quarters often led to interpersonal conflicts that were difficult to anticipate prior to moving in: “*it’s hard to tell a hundred percent how the roommate situation will be until I move in*” [Joy, Housed]. “*you can’t stand this person … what do you do then?*” [Jason, Unhoused]. Some even faced eviction due to conflicts with roommates (see Figure 3).

**Figure 4.**
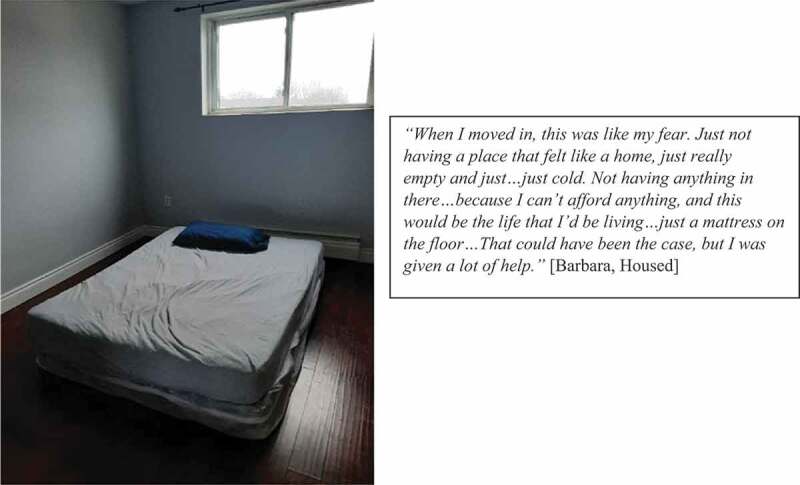
Creating home with limited resources.

**Figure 5.**
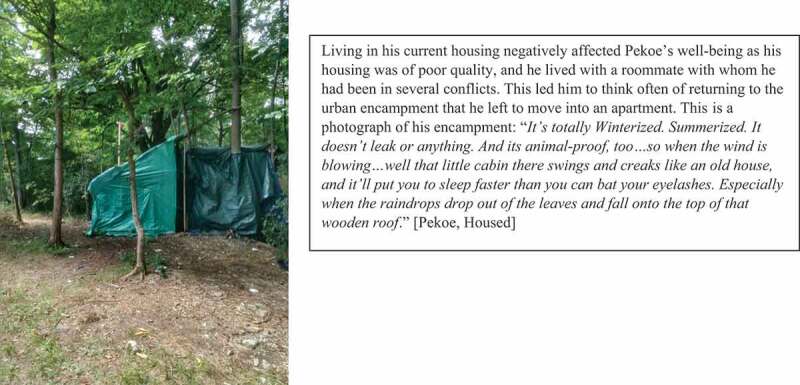
Reminiscing about returning to an encampment.

While some participants feared moving into housing alone with few resources for making their housing comfortable, others were able to secure the conditions that allowed their housing to feel like home with the help of friends, family, and local services (see Figure 4). Those who were unable to access such support after securing housing longed to return to encampments, shelters, or the street (see Figure 5).

#### Theme 2: finding and keeping ‘my people’

Participants described the importance of finding community as essential for supporting thriving following homelessness. This included both rekindling old relationships and building new ones beyond social networks developed in shelters and the street. Participants frequently discussed challenges they faced with being socially excluded during homelessness, a problem that followed them into their housing, and sometimes worsened without the social networks that surrounded them while they were unhoused: “*I don’t feel like I talk to anybody really in my day, and the people I meet and the places I go to and stuff. I don’t really have friends. I’ve lost my friends, it seems*” [Doc, Housed]. Experiences of disconnection were described as part of participants’ lives for a long time, causing them to give up on building relationships entirely: “*I don’t mind being alone … I’ve been alone forever*” [Cheech, Unhoused]. Participants also described giving up on building relationships that supported their mental well-being: “*I’ve gotten to the point where I feel why bother then?…I got too many disappointments … it’s hard to make friends with people that are good for you*” [Casey, Housed]. While they found developing these relationships to be challenging, they recognized how critical having these relationships were for both sustaining their housing and supporting well-being: “*The minute I lose that connection, that’s when I get unstable*” [Barbara, Housed].

**Figure 6.**
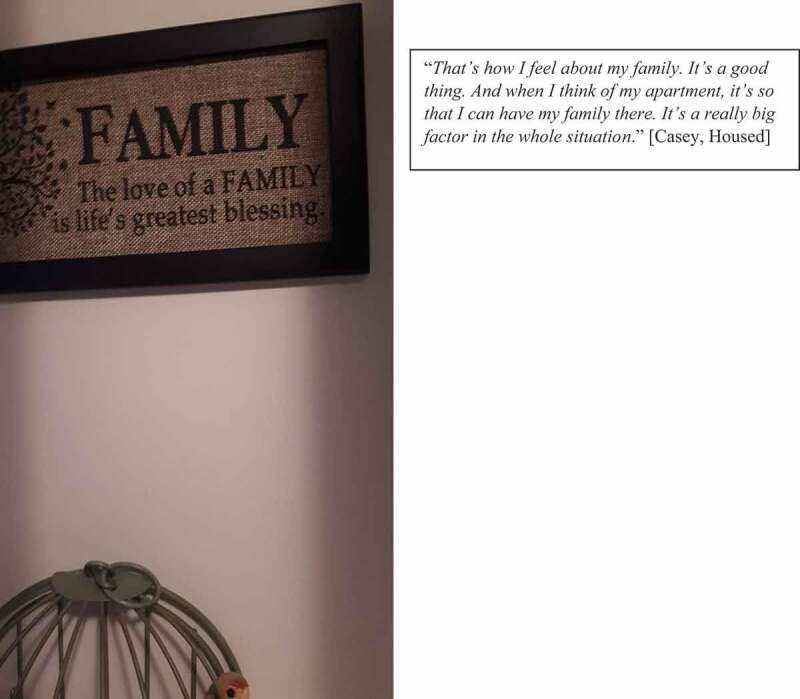
Family as supporting well-being and providing motivation for sustaining a tenancy.

Many participants tried to reconnect with friends and family after they were housed and saw these connections as an important motivator for creating home and sustaining their tenancies (see Figure 6). This included reconnecting with friends, children, parents and siblings with whom they had lost contact during homelessness due to embarrassment over being unhoused and struggles with ongoing substance misuse: “*I’m going through crystal withdrawal, like trying to get off crystal or whatever … that’s what I mean by not being healthy … [when in withdrawal] I don’t go around them … I just walk away from people*” [Ocean Breeze, Housed].

**Figure 7.**
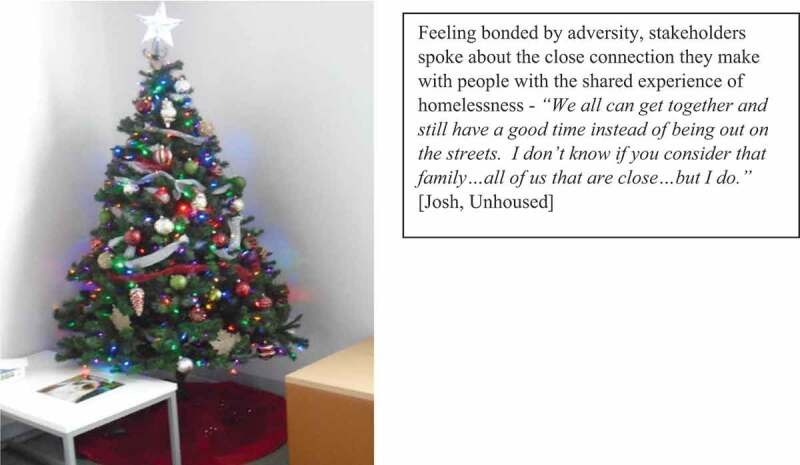
Creating family.

Some discussed how their family dynamics were problematic, leading them to believe that they needed to tread carefully when re-initiating these relationships. Others felt that reconnecting with family wasn’t an option for them at all and they created a sense of family with others who had also experienced homelessness in the past (see Figure 7).

Building relationships following homelessness was especially meaningful given that participants had lost so many relationships prior to and during homelessness, leaving them feeling alone and isolated before they moved into their housing: “*I had a lot of friends … be like I don’t even want to hang out with you anymore. Look at how messed-up you are*” [Peter, Unhoused]. When asked about what he needed to thrive following homelessness, one participant responded without hesitation: “*Just love really. Love … not making love. Not sexual. Just … being loved and loving another … the power of love … could be the most powerful force there is*” [Crispy, Unhoused].

#### Theme 3: meaningful activity as critical for thriving following homelessness

Participants emphasized that housing was a launching pad for engaging in a life that was meaningful, something that they found difficult to do while unhoused. For many, it took time for them to rediscover what they wanted to do with their time after the disruption of homelessness: “*I do tend to watch a lot more TV now than I used to, and I just started getting back into doing things like colouring that I haven’t done in a long time*” [Gabriella, Housed]. Some found ways to engage in activities that were meaningful while unhoused, and this continued after securing housing, which supported their mental health and connection to community: “*I love to cook … even when I wasn’t here, I was doing it. I’d go to my friends’ houses and cook … cause I love cooking … I’ve always been just whatever I want in the kitchen … I got a fully equipped kitchen, and I paid the storage to keep that … it brings … family together*” [Casey, Housed].

**Figure 8.**
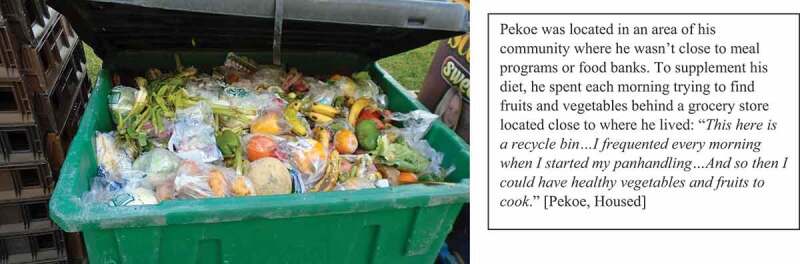
Spending time finding food.

Participants emphasized that opportunities to engage in meaningful activities was a crucial component of thriving following homelessness. When asked what he needed once housed to attain mental well-being, Josh responded: “*Something to do pretty much, it doesn’t really matter what it is*” [Josh, Unhoused]. While meaningful activity was emphasized by participants as essential for well-being, many lacked opportunities to engage in these activities both during and following homelessness. Their days continued to be consumed with survival activities including panhandling and finding food (see Figure 8), leading to boredom that threatened mental well-being: “*All’s we do is sit on the floor or sleep on the floor and do nothing … There’s nothing else for me. Watch TV … That’s it*” [Donny, Unhoused]. Boredom was also identified as a trigger for substance use:

*Y*ou can give someone a home, and you know, a full kitchen … but eventually boredom will kick in … For myself, it’s the worst trigger ever because I get in my own head … .and the bad thoughts come in and … then those thoughts start consuming my day and then consuming my week and before I know it … a relapse [Victor, Housed].

**Figure 9.**
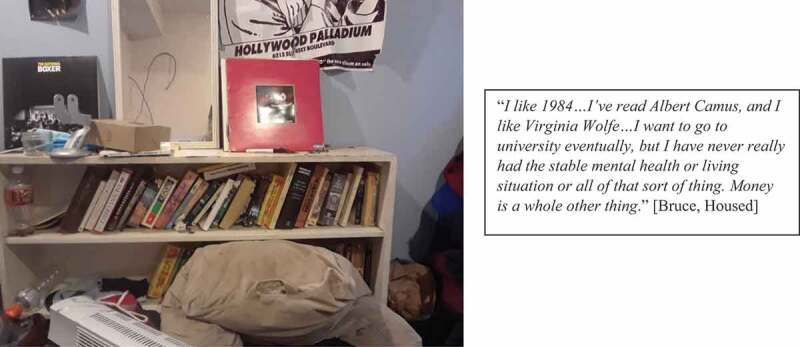
Wanting to return to school, but facing barriers to participation.

When able to engage in meaningful activities in their community, participants identified that these activities often served to connect them to the larger community and ultimately feel that they belonged: “*I belong because I’m able to help my community with … finding resources that they don’t know about … I’m able to tell them about it so they can access it*” [Michelle 2, Housed]. Several stakeholders voiced the desire to return to school or participate in paid employment. When asked about how she wanted to occupy her time once housed, Crimson emphasized “*a job, like employment is … number one*” [Crimson, Unhoused]. While a few stakeholders were able to engage in school or paid work once housed, others expressed that several barriers prevented them from achieving these goals including lack of financial resources to return to school and a lack of training opportunities and support to re-enter the workforce (see Figure 9): “*I’ve always wanted to go back to school … it’s hard for me because I can’t afford it these days*” [Suzie, Unhoused].

#### Theme 4: struggling to access mental health supports in the context of challenging circumstances

**Figure 10.**
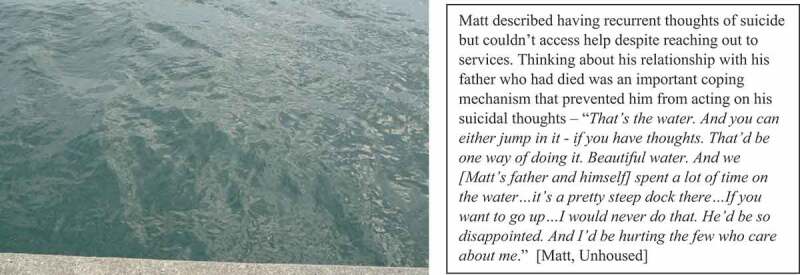
Coping when supports are unavailable.

Participants emphasized the need for increased access to mental health supports, which were seen to be lacking in their communities. Often, trauma preceded homelessness, and either contributed to the development of mental illness or substance use disorder or complicated a pre-existing mental health condition: “*I lost my daughter, and everything went downhill*” [Storeigh, Unhoused]. Homelessness itself was experienced as trauma, and securing housing enabled participants to have the emotional space to process these experiences, as it was impossible to process trauma while they were unhoused: “*I was always just surviving*” [Gavin, Housed]. Despite the presence of trauma, ongoing mental illness and substance misuse, participants discussed how mental health supports were unavailable or ineffective when they reached out: “*I felt like jumping off of a building, and literally, I was ready to do so … they just sent me out the door with information on a piece of paper … of like, how to cope with your mental anxiety … they weren’t taking me seriously at all*.” [James, Unhoused]. When such services were unavailable, participants coped with the presence of trauma and thoughts of suicide on their own by deriving hope and meaning from their immediate environments (see Figure 10).

Participants discussed at length how substance use challenges often sustained homelessness by negatively impacting their mental health: “*it just makes me a completely different person … I start making things up in my head*” [Crimson, Unhoused]. Relatedly, they acknowledged the importance of reducing their substance use to sustain their tenancies following homelessness: “*I gotta stop doing it … I gotta stop yelling at everybody … I know my health isn’t good … I’m doing crystal meth, and it’s a very dirty drug*” [Amber, Housed]. Others expressed the desire to continue using substances recreationally and didn’t see their use of substances as interfering with their ability to sustain their housing and thrive following homelessness: “*I’m not one of those people who is addicted to it … I may get it maybe once in a blue moon, when I have money*” [Suzie, Unhoused]. Ongoing substance use challenges in the environments of participants was described as chaotic and associated with increased exposure to trauma. One participant described finding a roommate who had died of an overdose: *“one day, I came home and found him dead … he had a needle in his arm and he was gone. So, that was rough for me*” [John, Unhoused] ; and “*there was fights. Some guy almost died. I got traumatized … cause I heard it all happen*” [Amber, Housed]. Most participants, however, identified that effectively managing their substance use through harm reduction approaches or by reducing or abstaining would enable them to more effectively manage in their daily lives and sustain their tenancies: “*I couldn’t afford to have to go back … it was the biggest thing that I got in check*” [Jason, Unhoused].

While harm reduction services were widely available in their communities, participants struggled to access substance use treatment services. Waitlists were several months long, and some participants were able to access such programs only by being especially persistent:

I met these guys at the meetings and they told me about [name of treatment centre] and I called every day for six weeks, five times a day. They eventually called me back and said: “I’m going to get you in sooner because I’m sick of hearing your voicemails”. It took six or seven weeks to get in. [Victor, Housed]

**Figure 11.**
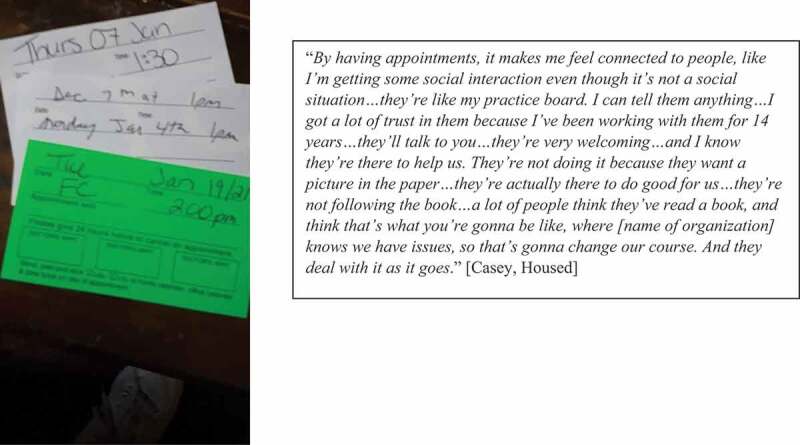
The meaning of having a connection with trauma-informed services.

Whether participants were accessing services for their mental health generally, or substance use disorders specifically, they were deeply appreciative of services that acknowledged their histories of trauma and sought to build meaningful and authentic relationships with them (see Figure 11). These relationships were seen to be pivotal in their journey towards recovery and tenancy sustainment following homelessness.

## Discussion

We conducted this study to identify, from the perspectives of persons with experiences of homelessness, what is needed to build on existing services to more effectively support individuals to thrive following homelessness in two communities in Ontario, Canada. While the findings of this study represent these two communities, the similarity in participant narratives across recruitment sites, and the findings of other literature (Boland et al., 2018; Oudshoorn, Van Berkum, et al., 2021) suggest that the conditions needed for thriving following homelessness identified in this study may be similar across communities. Our findings build on existing literature from the perspectives of service providers, organizational leaders, and persons with lived experience suggesting that homelessness does not end with securing a tenancy, but marks a new journey to finding home (Boland et al., 2018; Kerman & Sylvestre, 2020). Participants in this research indicated that they struggled to attain the necessary conditions for thriving following homelessness, and that the causes of these difficulties were primarily systemic. While it is commonly assumed that securing a tenancy results in increased access to resources for attaining psychosocial well-being, our findings suggest that some individuals live in a constant state of survival following homelessness that prevents this from occurring. Because participants’ basic needs for adequate shelter, food, and opportunities for social and meaningful activity engagement were unmet, some participants in the current study considered returning to homelessness, as the social and material conditions of their housing provided *less* comfortable living conditions than living shelters or on the street. Overall, our findings highlight the need for interventions that build on existing supports for supporting thriving, shifts in existing services to incorporate a relational, trauma-informed approach, and policies to address the systemic issues that prevent thriving following homelessness.

### Research implications

Our findings emphasize the particular importance of developing and evaluating interventions that build on existing supports to more effectively target thriving following homelessness. HF is the most commonly researched of these interventions, and a range of studies have demonstrated its effectiveness for improving indices of tenancy sustainment (Goering et al., 2014) and the cost effectiveness of HF compared with other approaches (Latimer et al., 2019). Across a range of studies, however, researchers have indicated that HF inconsistently demonstrates effectiveness for improving indices of psychosocial well-being including community and social integration, substance use, meaningful activity engagement, and symptoms of mental illness (Goering et al., 2014; C. A. Marshall et al., 2022, 2020; Quilgars & Pleace, 2016; Woodhall-Melnik & Dunn, 2015). This has led researchers to conclude that while HF needs to continue to be implemented, there is a need to build on the success of this approach by developing novel interventions that can be delivered in concert with HF to more effectively target psychosocial outcomes (C. A. Marshall et al., 2020, 2022). The findings of our research further emphasize this need. Researchers may consider co-designing such approaches alongside persons with lived experience, service providers, and organizational leaders to maximize relevance and uptake in their communities (Zamenopoulos & Alexiou, 2018). Further, researchers are encouraged to measure indices of thriving in the evaluation of these approaches to determine their effectiveness on both tenancy sustainment and indices of psychosocial well-being. While a range of standardized scales that measure indices of psychosocial well-being currently exist, there is a need to develop measures of thriving that are specific to the needs of individuals as they transition to housing following homelessness and that account for a range of indices in a single measure.

### Practice implications

Participants in the current study emphasized the need for resources and services following homelessness that would enable them to build social connections, engage in meaningful activities and support their mental well-being. Community integration has been a challenging outcome to target following homelessness (C. A. Marshall et al., 2020) likely due to the stigma of mental illness, substance use disorder and/or homelessness faced by this population (Dej, 2020; Forchuk & Csiernik, 2021). Further, participants in this study and in previous research have identified that meaningful activity engagement is a frequent challenge following homelessness (C. A. Marshall et al., 2018, 2020; Marshall, Gewurtz, et al., 2022), and a pathway to community integration (C. A. Marshall et al., 2022, 2022). Service providers may consider finding ways of intentionally targeting these constructs in the development of goals with persons who experience homelessness and integrate outcome measurement strategies that can evaluate the effectiveness of these approaches. Scholars in the interdisciplinary literature have highlighted the ways in which engagement in meaningful activity can facilitate relationship building through the concepts of “co-occupation” (Pickens & Pizur‐barnekow, 2009) and “collective occupations” (Ramugondo & Kronenberg, 2013). Identifying strategies that engage individuals leaving homelessness in meaningful activities that connect them with others in their community may be an important strategy for promoting community integration following homelessness. Few interventions, however, have been developed for the purpose of engaging individuals who experience homelessness in meaningful activity (Marshall, Easton, et al., 2022), or that engage individuals in meaningful activity to promote community integration, and such approaches need to be developed.

Participants in the current study emphasized the importance of accessing immediately available mental health and substance use services based on their individual needs, and the importance of their relationships with service providers in promoting recovery following homelessness. While the lack of available mental health and substance use services are related to systemic problems that exist at the policy level, practitioners are encouraged to use their practice knowledge to influence policy at the organizational, regional, and federal levels that limit access to needed services for persons with experiences of homelessness. Service providers should be aware of the importance that persons experiencing homelessness attribute to the services that they provide, a finding emphasized by participants in previous research (C. A. Marshall et al., 2022). Acknowledging the trauma histories that persons with histories of homelessness have experienced and integrating this knowledge in the delivery of care is a key approach for improving the effectiveness of services (Bransford & Cole, 2019; Hopper et al., 2010). A relational approach in which services are delivered to account for the trauma and attachment histories of persons who have experienced homelessness as a way of building trusting and authentic relationships has been emphasized by the participants in this study and has been promoted by existing practice frameworks (C. Marshall et al., 2020). Service providers may consider making concerted attempts to incorporate this knowledge in their practice in the support of individuals during and following homelessness.

### Policy implications

Mostly, the findings of this study build upon a significant body of literature that locates both causes and solutions to homelessness at the structural level. Participants in this study emphasized how they lived constantly without their needs met during and following homelessness, and that this placed them in ongoing precarity that perpetuated homelessness and placed their health at risk. Preventing and ending homelessness will be impossible without action on the part of policymakers to mandate an immediate and significant increase in public housing stock. Inaction on the part of policymakers to address the lack of investment in public housing in high-income countries has led to the significant and growing homelessness crisis in which we are currently embedded (Gaetz, 2010). Further, persons who experience homelessness are largely individuals who are living with disabilities (Fazel et al., 2014; Hossain et al., 2020). Participants in the current study all acknowledged living with a mental health condition or substance use disorder. Nearly 60% had been recognized by local governments to have a disability that was verified by a regulated health professional to qualify for disability-related social assistance (ODSP); yet most participants continued to struggle to meet their basic material needs and were forced into ongoing precarity due to an insufficient income provided by social assistance programs. Placing persons who are living with complex health histories in a state of ongoing need and denying them the right to safe and adequate housing is a serious disability rights violation and represents both the systematic exclusion of persons with disabilities from the right to well-being, and a failure of society to meet the needs of all citizens, regardless of the social locations they may occupy. In addition to increasing the availability of public housing, there is a need to introduce universal basic income (UBI) (Haagh, 2019) that is adjusted on an ongoing basis to enable all individuals to afford adequate housing and living costs based on the current cost of living. There is a strong and growing call by researchers and advocates for policymakers to implement economic policies including UBI, strategies for improving liveable wages, and enhancing social assistance programs (Carr et al., 2016; Haagh, 2019; Temple et al., 2019). Implementing some or all of these reforms is essential for addressing the growing income inequality that is increasingly fuelling the problem of homelessness in high-income countries worldwide.

Inequitable access to mental health and substance use supports was identified as a serious problem that prevented participants from attaining mental well-being following homelessness in this study. A lack of access to mental health services among persons experiencing homelessness is recognized as a serious problem across studies conducted in high-income countries (Canavan et al., 2012; Ramsay et al., 2019). Barriers to accessing such services in the present study included long waitlists, and a lack of responsiveness on the part of the mental health system. In previous research, inequitable access to mental health services has resulted from not providing services after “business hours,” the presence of stigma towards persons with histories of homelessness, and a lack of service coordination (Canavan et al., 2012). Poor access to such services is a serious problem in several high-income countries worldwide and is likely to further deepen disabilities already known to be prevalent among persons who experience homelessness (Fazel et al., 2014; Hossain et al., 2020). Policymakers need to account for the limited access of persons leaving homelessness to mental health and substance use services by designing policies that increase the availability of such supports specifically for this population. Such policies have the potential to support persons leaving homelessness to attain well-being and address any challenges with substance use that interfere with their mental health and ability to sustain their tenancies, a problem identified in the current study and in previous research.

### Limitations

As a qualitative study, the findings of this research are meant to be transferred, rather than generalized to persons who experience homelessness in high-income countries. As such, the views of participants in this research cannot be interpreted as inclusive of the views of all persons leaving homelessness. Our findings also do not represent the experiences of leaving homelessness for persons living in low- to middle-income countries. As we only included participants with experiences of mental illness and substance use disorder in this study, our findings represent the perspectives of individuals transitioning from homelessness who represent this sub-group. Further, the majority of participants that we interviewed in this research were White, heterosexual, and cis-gendered. Participants representing other races, persons identifying as 2SLGBTQ+, and non-binary are not well represented by the findings of this research, and more research exploring the experiences of transitioning from homelessness for these under-represented groups is needed. It should also be noted that we conducted this research during the COVID-19 pandemic, and we recognize that the experiences of participants may have been influenced by public health policies implemented during the time of our interviews. Finally, as one of our interviews was only nine minutes in duration, the lack of depth attained in the context of this interview should be seen as a limitation of the findings of our research.

## Conclusion

To prevent ongoing homelessness in high-income countries, there is a need for providing individuals with access to resources that lead to improvements in their lives beyond shelters and the street. Our findings suggest that this is not happening for many individuals following homelessness. Providing good quality housing that is affordable, an adequate income, and access to services that support mental health recovery are essential to fulfilling this aim. Without having these needs met, individuals leaving homelessness are asked to forego some basic needs (e.g., social networks, food) that were met in shelters and the street to attain others (a private dwelling) when housed. This is an impossible position in which to place individuals as they leave homelessness, and there is a need for building a system that regards “thriving” rather than “tenancy sustainment” as the end goal. The focus of our research on identifying the conditions needed for thriving following homelessness is a novel contribution in that it represents a shift in thinking about homelessness prevention by moving beyond targeting tenancy sustainment as a primary indicator of what it means to end homelessness. Instead, this research challenges researchers, practitioners, and policymakers to consider more than just tenancy sustainment in determining the success of interventions, programs and policies developed to prevent and end homelessness in high-income countries worldwide.
